# Identification of *AMH* and *AMHR2* Variants Led to the Diagnosis of Persistent Müllerian Duct Syndrome in Three Cases

**DOI:** 10.3390/genes13010159

**Published:** 2022-01-17

**Authors:** Yang Liu, Sida Wang, Ruzhu Lan, Jun Yang

**Affiliations:** 1 Department of Neurology, Tongji Hospital, Tongji Medical College, Huazhong University of Science and Technology, Wuhan 430030, China; happyliuyang2011@163.com; 2 Department of Urology, Tongji Hospital, Tongji Medical College, Huazhong University of Science and Technology, Wuhan 430030, China; sidaw@yahoo.com (S.W.); rzlan@tjh.tjmu.edu.cn (R.L.); 3 Institute of Urology, Tongji Hospital, Tongji Medical College, Huazhong University of Science and Technology, Wuhan 430030, China

**Keywords:** PMDS, cryptorchidism, Müllerian remnants, *AMH*, *AMHR2*, genetic analysis

## Abstract

Persistent Müllerian duct syndrome (PMDS) is a rare autosomal recessive disorder of sexual development in males, defined by the presence of Müllerian remnants with otherwise normal sexual differentiation. Mutations in anti-Müllerian hormone (*AMH*) and AMH receptor type 2 (*AMHR2*) genes are the main causes of PMDS. In this study, we performed molecular genetic analysis of 11 unrelated cryptorchidism patients using whole-exome sequencing and classified the variants. Three of the 11 patients had biallelic mutations in *AMH* or *AMHR2*. Case 1 carried a homozygous 4-bp deletion; c.321_324del:p.Q109Lfs*29 in exon 1 of *AMH* (NM_000479 transcript), which is a frameshift mutation, leading to the loss of function of AMH. Case 2 carried compound heterozygous mutations; c.494_502del (p.I165_A168delinsT) in exon 4 and g.6147C>A of *AMHR2* (NM_001164690 transcript). Case 3 carried compound heterozygous mutations; c.G1168A (p.E390K) in exon 9 and c.A1315G (p.M439V) in exon 10 of *AMHR2* (NM_001164690 transcript). All three patients were admitted due to azoospermia- and oligospermia-caused infertility. They were furtherly diagnosed with PMDS, as pelvic magnetic resonance imaging revealed the presence of Müllerian remnants. Our study suggests that PMDS and genetic analysis should be considered during the differential diagnosis of cryptorchidism.

## 1. Introduction

Persistent Müllerian duct syndrome (PMDS, MIM#261550) is a rare autosomal recessive disorder of sexual development that affects males; it is defined by the presence of Müllerian remnants (i.e., fallopian tubes, uterus, and upper vagina), normal karyotype (46, XY), and male external genitalia [1]. In 1939, Nilson et al. diagnosed PMDS in a male infant for the first time [2]. Anti-Müllerian hormone (AMH) is normally secreted by the Sertoli cells of the testis. Müllerian structures completely vanish as a result of the effects of AMH during the tenth week of fetal development, which mediates normal sexual differentiation in males [3,4], while Müllerian ducts differentiate into fallopian tubes, uterus, and upper vagina in females in the absence of AMH secretion or activity [5].

Mutations in AMH and AMH receptor type 2 (*AMHR2*) genes are the main causes of PMDS [6]. About 88% of PMDS cases are caused by compound heterozygous or homozygous mutations in *AMH* or *AMHR2*, while the remaining 12% of cases that cannot be genetically diagnosed are categorized as idiopathic [7]. There are no differences in the phenotypic manifestations of PMDS caused by mutations in these two genes. However, patients with *AMH* mutations usually show low or undetectable serum AMH levels compared to normal or elevated serum AMH levels found in cases with *AMHR2* mutations [6].

The main clinical manifestation of PMDS is undescended testis, including impalpable undescended testis on one side with palpable undescended testis or inguinal hernia, impalpable undescended testis on both sides, or transverse ectopia [8,9]. The Müllerian duct is attached to the testicles and prevents them from descending to the scrotum from the inguinal rings.

PMDS in cryptorchidism patients are easily ignored as most previous cases were diagnosed incidentally when Müllerian remnants were detected during ultrasonographic evaluation of undescended testes or inguinal hernia. If cryptorchidism patients with PMDS are not differentially diagnosed, they might have chance to develop health problems. Furthermore, as most PMDS patients were reported to carry genetic variants in *AMH* or *AMHR2* gene, lack of differential diagnosis of cryptorchidism patients will increase the genetic risks in the second generation to a certain extent.

This study reports genetic analysis of eleven cryptorchidism patients, with the results of clinical and molecular genetic of three differentially diagnosed PMDS.

## 2. Materials and Methods

### 2.1. Patients and Samples

We randomly selected 11 unrelated patients who were diagnosed with cryptorchidism and were treated for male infertility at the Reproduction Center of Tongji Hospital in Wuhan. Genetic analysis and pelvic magnetic resonance imaging (MRI) were performed for all patients. Peripheral blood samples (2 mL each) of all patients were collected. Written informed consent was obtained from all the participants. This research was approved by the Research Ethics Committee of Tongji Hospital (approval no. TJ-IRB20211138).

### 2.2. Genetic Analysis

Genomic DNA was extracted from blood samples using the TIANamp Blood DNA Extraction Kit (TIANGEN Biotech Co., Ltd., Beijing, China). Whole-exome sequencing was performed using IDT xGen Exome Research Panel V1.0 (Integrated DNA Technologies, Coralville, Iowa, IA, USA). The quantity of sequencing library was assessed by Qubit 2.0 fluorometer (Thermo Fisher Scientific, Cleveland, OH, USA). The quality and size of the libraries were assessed by 2100 Bioanalyzer High Sensitivity DNA Assay (Agilent Technologies, Palo Alto, CA, USA). For next-generation sequencing, the qualified libraries were subjected to 2 × 150-bp paired-end sequencing on the Illumina NovaSeq platform (Illumina, San Diego, CA, USA). FASTQ files were aligned with the human reference genome (hg19/ GRCh37), using BWA v0.7.13. Variants (single nucleotide variants and indels) were genotyped from recalibrated BAM files using GATK 4.0 and were annotated using ANNOVAR against multiple databases, including HGVS variant description, population frequency, disease or phenotype, and variant functional prediction. Variants were classified as pathogenic, likely pathogenic, variants of unknown significance (VUS), likely benign, or benign, according to the American College of Medical Genetics (ACMG) guidelines. Copy number variants were identified using the DNA copy R package, filtered and classified by ACMG guidelines, and manually checked using the Integrative Genomics Viewer.

## 3. Results

Among the 11 cryptorchidism patients, three had mutations in either *AMH* or *AMHR2*, and these three patients were diagnosed with PMDS because pelvic MRI revealed the presence of Müllerian remnants. The results of clinical and molecular genetic analyses of these three PMDS patients were reviewed in detail.

### 3.1. Case 1

Two years ago, a 29-year-old male patient, whose wife was unable to conceive after six months of marriage, underwent routine semen analysis in a local hospital. He was diagnosed with azoospermia after hormone analysis; its results are as follows: FSH level: 22 mIU/mL; LH level: 6.02 mIU/mL; E2 level: 287 pmL/L; and T level: 11.1 nmol/L. He was considered to have non-obstructive azoospermia, and no sperms were found after microdissection testicular sperm extraction (micro-TESE). He then visited our medical center for another micro-TESE. Ten years ago, the patient had impalpable undescended testis on both sides, with bilateral testicles descending to the bilateral inguinal area; bilateral laparoscopic orchiopexy was performed in 2010. After the surgery, his left testicle descended into the scrotum, while the right testicle did not. No sperms were found in the semen during the following two years. Pelvic MRI in a local hospital revealed that the left testicle was in the scrotum and the right testicle was in the inguinal area. There were no other obvious anomalies. It is noteworthy that this patient was born in a consanguineous family.

**Figure 1:**
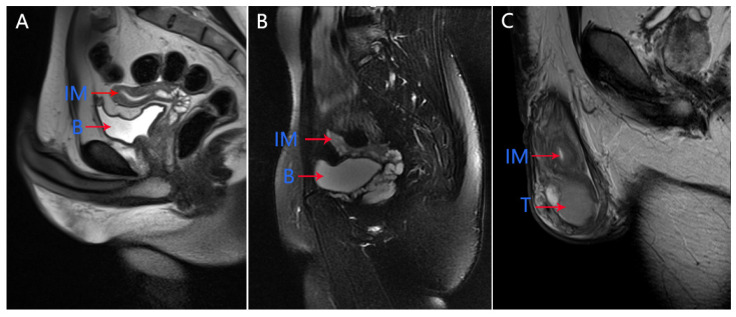
Pelvic magnetic resonance images of the three cases. Pelvic magnetic resonance images show Müllerian remnants in cases 1 (**A**), 2 (**B**), and 3 (**C**). IM, immature uterus; B, bladder; T, testis.

**Figure 2:**
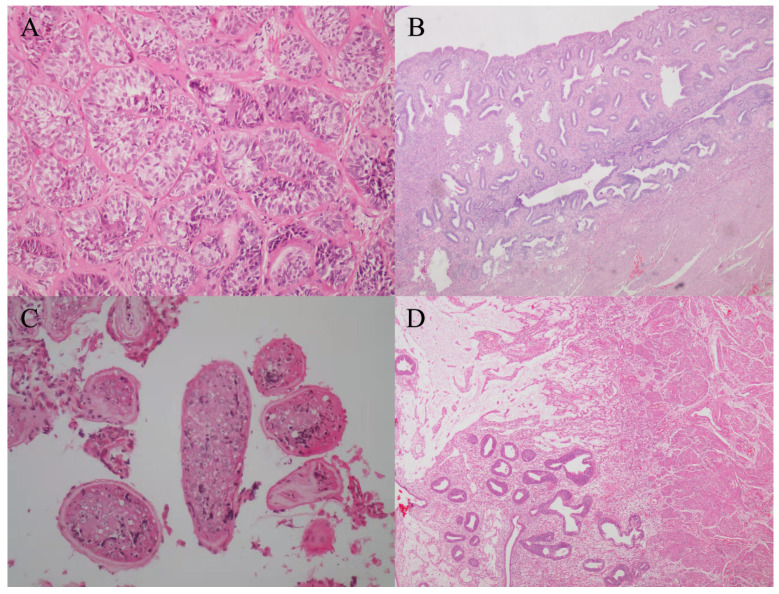
Histopathological staining. Histopathological analysis confirmed the organs of case 1 as testes (**A**, ×200 magnification) and uteri (**B**, ×40 magnification), and the organs of case 2 as testes (**C**, ×200 magnification) and uteri (**D**, ×40 magnification).

In our medical center, physical examination showed that the left testicle was palpable (10 mL) and the right testicle was impalpable. Penis and secondary sexual characteristics were well developed. The patient had the normal 46, XY karyotype. The serum AMH level was 0.06 ng/mL, which was significantly below the lower limit of the normal range (adult male: 2.04–19.22 ng/mL). Pelvic MRI showed the presence of uterus and upper two-thirds of the vagina at normal positions (Figure 1A). Micro-TESE was performed again, but no sperms were observed. Laparoscopic removal of Müllerian remnants was performed a week after the patient underwent micro-TESE. The patient was histologically confirmed to have PMDS (Figure 2A,B). Pathological examination of the patient revealed the presence of a normal uterus with uterine fibroids (Figure 2B).

### 3.2. Case 2

Two years ago, a 27-year-old male patient, whose wife was unable to conceive after six months of marriage, came to our medical center for routine semen analysis. He was diagnosed with azoospermia after hormone analysis; its results are as follows: FSH level: 38.26 mIU/mL; LH level: 9.93 mIU/mL; E2 level: 438 pmL/L; and T level 2.7 nmol/L. He was considered to have non-obstructive azoospermia. Pelvic MRI in a local hospital revealed that the right testicle was in enterocoelia with left testicle in bilateral inguinal area, and bilateral laparoscopic orchiopexy was performed in 2017. After the surgical procedure, no sperms were found in his semen. Therefore, he visited our medical center for micro-TESE. The patient had a left laparoscopic hernia repair when he was 5 years old, and both right and left testicular retraction occurred successively.

In our medical center, physical examination of the patient showed that both sides of the testicle were palpable (6 mL). Penis and secondary sexual characteristics were well developed. The patient had the normal 46, XY karyotype. Serum AMH level was 3.21 ng/mL. Pelvic MRI revealed an immature uterus at the normal position (Figure 1B). Micro-TESE was performed and sperms were observed; Müllerian remnants were removed laparoscopically. Seminal vesicles and uteri were observed through transurethral seminal vesiculoscopy combined with laparoscopy, and the uterus was found to be connected to the urethra, while one of the openings was found in the verumontanum. The patient was histologically confirmed to have PMDS (Figure 2C,D).

### 3.3. Case 3

Six months ago, a 33-year-old male patient, whose wife could not conceive after six months of marriage, came to our medical center for routine semen analysis. Semen analysis showed oligospermia with no active or morphologically normal sperms. The results of hormone analysis are as follows: FSH level: 8.22 mIU/mL; LH level: 3.81 mIU/mL; E2 level: 24.41 pmL/L; and T level: 4.14 nmol/L. The patient was considered to have severe oligospermia. As per pelvic MRI analysis, right testicle was not detected and his left inguinal area was broadened. An internal mixed signal was detected throughout the left scrotum. The left testis suffered from oppression.

The patient had the normal 46, XY karyotype. Serum AMH level was 7.72 ng/mL. Pelvic MRI revealed the presence of an immature uterus at the normal position (Figure 1C).

### 3.4. Genetic Analysis

NGS sequencing revealed mutations in *AMH* or *AMHR2* in the three patients (Table 1). A homozygous 4-bp deletion, c.321_324del:p.Q109Lfs*29, was detected in exon 1 of *AMH* (NM_000479 transcript) in Case 1. This is a frameshift mutation and leads to the loss of function of the AMH. In both cases 2 and 3, two compound heterozygous mutations in *AMHR2* were detected. Case 2 carried compound heterozygous mutations: c.494_502del (p.I165_A168delinsT) in exon 4 and g.6147C>A of *AMHR2* (NM_001164690 transcript). Case 3 carried compound heterozygous variations: c.G1168A (p.E390K) in exon 9 and c.A1315G (p.M439V) in exon 10 of *AMHR2* (NM_001164690 transcript). According to the ACMG guidelines, all mutations except for g.6147C>A were classified as likely pathogenic. None of the three patients agreed to undergo further genetic analyses.

## 4. Discussion

PMDS is a rare autosomal recessive disorder of sexual development, defined by the presence of Müllerian remnants (fallopian tubes, uterus, and upper vagina) in males. There have been three anatomical variations of PMDS, of which bilateral undescended intra-abdominal testes account for the majority (approximately 60% to 70%) of PMDS cases, and hernia uteri inguinalis (approximately 20% to 30%) and transverse testicular ectopia (approximately 10%) account for the remaining PMDS cases [8,10]. Most PMDS cases are diagnosed incidentally during surgery for cryptorchidism or inguinal hernia, and the Müllerian remnants may stem the testes against descending into the scrotum.

The occurrence rate of PMDS is low, with over 200 cases reported to date. A review on the basis of 157 personal cases showed that *AMH* and *AMHR2* variants were detected in 40.4% and 45.7% of cases, respectively, and no mutations in *AMH* or *AMHR2* were detected in the remaining 13.9% [7]. It has been reported that mutations are scattered across the five exons of *AMH*. Picard et al. indicated that out of the 64 different mutations detected in *AMH*, most mutations were identified in exons 1, 2, and 5 [7]. In addition, this variant was frameshift and led to the loss of function of the AMH protein. Most of the identified mutations in *AMH* are missense mutations, with only few frameshift mutations [11,12]. We speculate that c.321_324del:p.Q109Lfs*29 may be a novel frameshift variant of *AMH*.

Most mutations in *AMHR2* were detected in exon 10 [7]. In a study of 11 cases from six unrelated Turkish families, seven different mutations in exon 1, 2, 10, and 11 of *AMHR2* were detected [13]. Fang et al. identified compound heterozygous mutations of c.1387C>T (p.R463C) and c.1219C>T (p.R407X) in exons 9 and 10, respectively, of *AMHR2* (NM_020547.2) in two brothers who had a history of bilateral cryptorchidism with orchidopexy as well as infertility due to azoospermia [14]. In our study, compound heterozygous mutations in exon 9 (c.G1168A (p.E390K)) and exon 10 ((c.A1315G (p.M439V)) of *AMHR2* (NM_001164690 transcript) were identified in case 3, who had a history of infertility due to oligospermia. Compound heterozygous mutations in c.494_502del (p.I165_A168delinsT) in exon 4 and g.6147C>A of *AMHR2* (NM_001164690 transcript) were identified in case 2, who had a history of left laparoscopic hernia repair and bilateral laparoscopic orchiopexy as well as infertility due to azoospermia. These findings suggest that PMDS and genetic analysis should be considered during the differential diagnosis of cryptorchidism. For patients with suspected PMDS, preoperative AMH hormone examination, genetic analysis, and MRI after informing the imaging physician of possible uterine remnants can improve the diagnosis rate of PMDS. In this study, 11 randomly selected patients with cryptorchidism were diagnosed with PMDS. The actual morbidity could be higher than expected.

Because of the increasing number of reports of Müllerian malignancies [15,16,17], preoperative diagnosis is very important. In this study, two of the three patients (cases 1 and 2) underwent pelvic MRI before surgery in a local hospital, but the presence of a uterus was not reported in either of them. This suggests that the diagnosis of PMDS on the basis of imageological examination before surgery is difficult. PMDS is a rare condition, and most radiologists lack relevant experience for its diagnosis. Therefore, even surgical diagnosis of PMDS could be missed (for cases 1 and 2). Hence, the surgeon’s experience is extremely important. In case 2, we used transurethral seminal vesiculoscopy combined with laparoscopy to detect the seminal vesicle and uterus. In addition, we found that the uterus was connected to the urethra and one of its openings was present in the verumontanum. Prior to our surgery, there had been some reports of the uterus found to be connected to the urethra during urography. We surgically confirmed this condition in our patient.

In the past 50 years, PMDS has been linked to infertility and testicular malignancy, and most studies have suggested leaving the Müllerian remnants in situ because the remnants were considered difficult to dissect and there was no evidence that PMDS patients have a higher risk of cancer than normal people [1,7,18]. Most of these studies suggested that the main purpose of surgery is to retain fertility and prevent testis cancerization. No sperms in semen was observed in cases 1 and 2 of this study, and the micro-TESE success rate was 50%, which was similar to the rate of surgical sperm retrieval in men with azoospermia without PMDS [19]. Some reports indicated that the incidence of malignant change in these testes was approximately 5% to 18% [20,21], which was also the same as that in men with cryptorchism without PMDS. However, another study reported that about 3.1% to 8.4% of male patients with PMDS will develop malignancy [16]. The development of technology such as laparoscopy provides a higher definition magnification of images, which allows us to accurately and easily separate anatomical structures. We believe that the removal of the uterine remnants of patients with PMDS would be a safe option. As we wanted to determine a way to diagnose PMDS without surgery, a retrospective review of these cases was very helpful in distinguishing between cryptorchidism and PMDS, and avoiding misdiagnosis of PMDS.

## 5. Conclusions

We diagnosed three patients with PMDS from 11 randomly selected unrelated patients with cryptorchidism. Five mutations in *AMH* and *AMHR2* of the three PMDS patients were identified. Our study suggests that PMDS and genetic analysis should be considered during differential diagnosis of cryptorchidism.

## Figures and Tables

**Table 1:** The detected gene mutations of patients.

Case	Gene	Variation (DNA)	Variation (Protein)	Location	Zygocity	ACMG Classification
case1	*AMH*	NM_000479: c.321_324del	p.Q109Lfs*29	exon1	homozygous	Likely Pathogenic
case2	*AMHR2*	NM_001164690: c.494_502del	p.I165_A168delinsT	exon4	compound heterozygous	Likely Pathogenic
case2	*AMHR2*	NM_001164690:g.6147C>A	-	-	compound heterozygous	Uncertain significance
case3	*AMHR2*	NM_001164690:c.G1168A	p.E390K	exon9	compound heterozygous	Likely Pathogenic
case3	*AMHR2*	NM_001164690:c.A1315G	p.M439V	exon10	compound heterozygous	Likely Pathogenic

## Data Availability

The data presented in this study are available in article.
